# CDK4/6 inhibitor resistance: A bibliometric analysis

**DOI:** 10.3389/fonc.2022.917707

**Published:** 2022-12-01

**Authors:** Jiayuecheng Pang, Hengyu Li, Yuan Sheng

**Affiliations:** Department of Breast and Thyroid Surgery, Changhai Hospital, Naval Military Medical University, Shanghai, China

**Keywords:** CDK4/6 inhibitor, resistance, bibliometrics, breast cancer, research frontier

## Abstract

**Background:**

Cyclin-dependent kinases (CDKs) 4/6 inhibitors are a type of cell cycle regulation that prevents cell proliferation by blocking retinoblastoma protein (Rb) phosphorylation in the G1 to S phase transition. CDK 4/6 inhibitors are currently used mainly in patients with hormone receptor-positive/human epidermal growth factor receptor 2 (HER2) negative breast cancer in combination with endocrine therapy. However, primary or acquired resistance to drugs severely affect drug efficacy. Our study aims at summarizing and visualizing the current research direction and development trend of CDK4/6 inhibitor resistance to provide clinicians and research power with a summary of the past and ideas for the future.

**Methods:**

The Web of Science Core Collection and PubMed was searched for all included articles on CDK4/6 inhibitor resistance for bibliometric statistics and graph plotting. The metrological software and graphing tools used were R language version 4.2.0, Bibliometrix 4.0.0, Vosviewer 1.6.18, GraphPad Prism 9, and Microsoft Excel 2019.

**Results:**

A total of 1278 English-language articles related to CDK4/6 inhibitor resistance were included in the Web of Science core dataset from 1996-2022, with an annual growth rate of14.56%. In PubMed, a total of 1123 articles were counted in the statistics, with an annual growth rate of 17.41% Cancer Research is the most included journal (102/1278, 7.98%) with an impact factor of 13.312 and is the Q1 of the Oncology category of the Journal Citation Reports. Professor Malorni Luca from Italy is probably the most contributing author in the current field (Publications 21/1278, 1.64%), while Prof. Turner Nicholas C from the USA is perhaps the most authoritative new author in the field of CDK4/6 inhibitor resistance (Total Citations2584, M-index 1.429). The main research efforts in this field are currently focused on Palbociclib and Abemaciclib. Studies on drug resistance mechanisms or post-drug resistance therapies focus on MEK inhibitors and related pathways, PI3K-AKT-MTOR pathways or inhibitors, EGFR-related pathways, EGFR inhibitors, TKI inhibitors, MAPK pathways and inhibitors, and so on.

**Conclusion:**

This study provides researchers with a reliable basis and guidance for finding authoritative references, understanding research trends, and mining research neglect directions.

## Introduction

The human body has a cell cycle that regulates the process of cell proliferation, which is the entire process that cells undergo from the end of the first division to produce new cells to the end of the second division. In the regulation of the cell cycle process, cell cycle proteins and their chaperone kinases, cyclin-dependent kinases (CDKs), play an important role. CDKs are serine/threonine protein kinases whose activities are influenced by inhibitory factors and signaling pathways. In malignant tumors, CDKs are usually more active, thus generating a sustained proliferative signal (1, 2). Therefore, inhibition of CDKs has a unique advantage for curbing tumor progression. CDK4/6 binds to cyclin D to regulate the cell cycle from the G1 phase to the S phase *via* the R-point. The R-point mainly controls the duration of the G1 phase. With the passage of this point, the cell can complete the rest of the cell cycle at a normal rate independent of external conditions. Cyclin D-CDK4/6 complex binding to p21 or p27 generates a holoenzyme that phosphorylates retinoblastoma proteins (Rb) (3–5), while inducing partial derepression of E2F and expression of cell cycle protein E (cyclin E) (6). Cyclin E binds to CDK2 to hyperphosphorylate Rb, and eventually, E2F is separated from Rb and activated. The G1/S phase transition is driven by the E2F transcription factor. CDK4/6 inhibitors can inactivate cyclin D-CDK4/6 from G1 to S phase and block Rb phosphorylation (7). In addition, CDK4/6 inhibitors play an important role in regulating metabolism and tumor microenvironment (8).

CDK4/6 inhibitors are currently used to treat hormone receptor (HR) positive/human epidermal growth factor receptor 2 (HER2) negative breast cancer. The highly selective CDK4/6 inhibitors that have been approved by the U.S. Food and Drug Administration include palbociclib, abemaciclib, and ribociclib (2, 9–13). All three drugs were involved in trials in combination with non-steroidal aromatase inhibitors. The PALOMA-2 trial showed a median progression-free survival of 24.8 months in the palbociclib group compared to the placebo group (14.5 months in the placebo group, HR: 0.58, p<0.001) (11). The MONALEESA-2 trial showed a median progression-free survival of 25.3 months in the Ribociclib group (16.0 months in the placebo group, HR: 0.57, p<0.001) (12). The median progression-free survival in the Abemaciclib group in the MONARCH 3 study had not been reached (14.7 months in the placebo group, HR: 0.54, p<0.001) (13). All these drugs are approved for use in combination with nonsteroidal aromatase inhibitors in patients with metastatic hormone receptor +/HER2- breast cancer. Also, given the results of the CDK4/6 inhibitors in combination with fulvestrant in the trials PALOMA-3, MONALEESA-3, and MONARCH 2 for median progression-free survival (HR 0.46,0.60,0.55, respectively), three drugs were approved for use in combination with fulvestrant in patients with tumor progression after first-line endocrine therapy (14–16). The combination of CDK4/6 inhibitors did improve the prognosis of patients compared to endocrine therapy alone, but the inevitable problem was that 25%–35% of patients did not respond, and almost all patients eventually acquired resistance (17). For acquired resistance, authoritative studies have shown that the chronic loss of Rb is one of the important mechanisms leading to resistance to palbociclib, one of the CDK4/6 inhibitors. Furthermore, FAT1 loss, which is important as well, led to marked elevations in CDK6, the suppression of which restored sensitivity to CDK4/6 inhibitors. Other studies also revealed some of the causes of acquired resistance like activation of oncogenic growth factor signaling pathways, and changes in metabolic function (18–20). Whether these causes can be responded to in clinical applications remains to be proven by numerous studies.

Research on CDK4/6 inhibitor resistance is in full swing, but a detailed analysis of the existing research publications have not yet been performed. The study of published articles on CDK4/6 inhibitor resistance provides insight into current research hotspots and subjects in the field, and identifies emerging developments or under-researched parts, thereby guiding researchers’ research directions and gauging future research developments. This article presents a bibliometric analysis of articles related to CDK4/6 inhibitor resistance included in the Web of Science Core Collection from 1996 to 2022 and in PubMed from 1993 to 2022. The aim is to identify trends in the publication of common research topics, the focus of published journals, and the authorship of dominant countries.

## Methods

### Access to the original information

Data in this study were obtained from PubMed and the core collection of Web of Science, a database of Clarivate Analytics, and were updated on July 31, 2022. The citation indexes of Web of Science included SCI-EXPANDED, SSCI, AHCI, CPCI-S, CPCI-SSH, BKCI-SSH, ESCI, CCR-EXPANDED, and IC. Due to the textual habits of different authors and the limitations of database search engines on symbol search, the search strategy in this study used ALL = (cdk4 6 inhibitor) OR ALL = (cdk 46 inhibitor) OR ALL=(cdk4 inhibitor) OR ALL=(cdk6 inhibitor) to represent the search range of CDK4/6, combined drug resistance-related search range ALL=(resistance) OR ALL=(resistant). The exact search was not selected. A total of 1286 results were obtained from the Web of Science Core Collection, with 1278 articles (99.38%) in English, 6 articles in French, 1 article in German, and 1 article in Japanese in the language of publication. In PubMed, there are 1123 papers in English, 4 in French, 3 in Chinese, 2 in German, 2 in Japanese, and 1 in Hungarian. Only articles in English were selected, spanning 1993-2022. Results of Web of Science were exported as plain text files with full records and cited references and results of PubMed were exported as the format of ‘PubMed’. Since the PubMed database does not provide article citation data, bibliometric content involving citations was calculated using the Web of Science database only. The journal impact factors are extracted from Journal Citation Reports 2021 published by Clarivate Analytics.

### Data analysis and graph acquisition

The bibliometrix package (version 4.0.0) was installed in R 4.2.0using R studio (version 2022.02.2 + 485). Biblioshiny is a software that provides a web-based graphical interface in the bibliometrix package, which was used to import the data and perform bibliometric analysis (21). The data were also imported into Vosviewer (version 1.6.18) to generate network diagrams. The network was created using bibliographic data, and different types of analysis were selected according to the content of the analysis, such as co-authorship, co-occurrence, citation, etc. Full counting was used for each count, and articles containing a large number of authors were not excluded. In the process of exploring the mechanism and therapeutic content of CDK46 inhibitor resistance, we selected only Clinical Study, Clinical Trial, Controlled Clinical Trail, Randomized Controlled Trial, and Research Support from PubMed, and Article plus Meeting Abstract from Web of Science for statistical purposes. Reviews are not included in the statistics. Only the frequency of words is counted. Correlation is calculated using the Pearson correlation coefficient. Microsoft Excel 2019 and GraphPad Prism 9.3.1 were used to organize the data tables and modify the graphs.

## Results

### Overview

**Figure 1:**
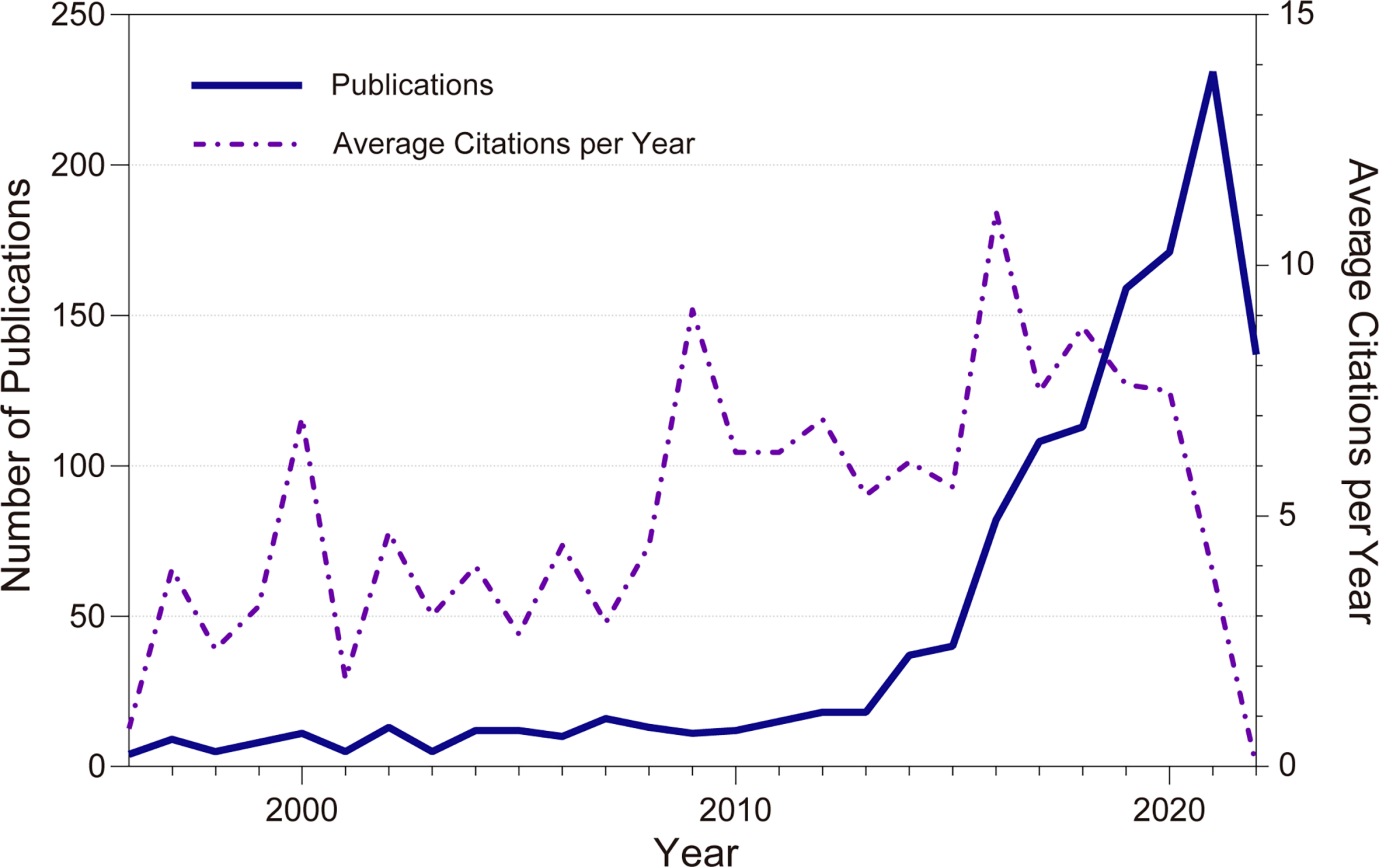
Annual publications of resistance to CDK4/6 inhibitors in Web of Science Core Collection.

A total of 1278 English-language articles related to CDK4/6 inhibitor resistance were included in the Web of Science Core Collection from 1996 to 2022. Among them, 911 articles (71.28%), 238 reviews (18.62%), and 91 meeting abstracts (7.12%) were published. From 4 articles published in 1996 to 231 articles in 2021, the annual growth rate of scientific article output is 14.56%. (**Figure 1**) While in PubMed, a total of 1123 articles were counted in the statistics, including 777 articles, 186 reviews, and no meeting abstracts included. The annual growth rate is 17.41%. There were three citation peaks in 16 years, in 2000, 2009, and 2016. In 2000, Sherr, CJ’s discussion on the cell cycle was the theoretical basis for CDK46 inhibitors (22). In 2009, findings of Palbociclib depicted a bright future for CDK46 inhibitors (23). The publication of the promising results of Fulvestrant plus palbociclib, Ribociclib, and the progressive emergence of CDK46 inhibitor resistance in 2016 have attracted widespread attention (12, 24).

### Journals

**Figure 2:**
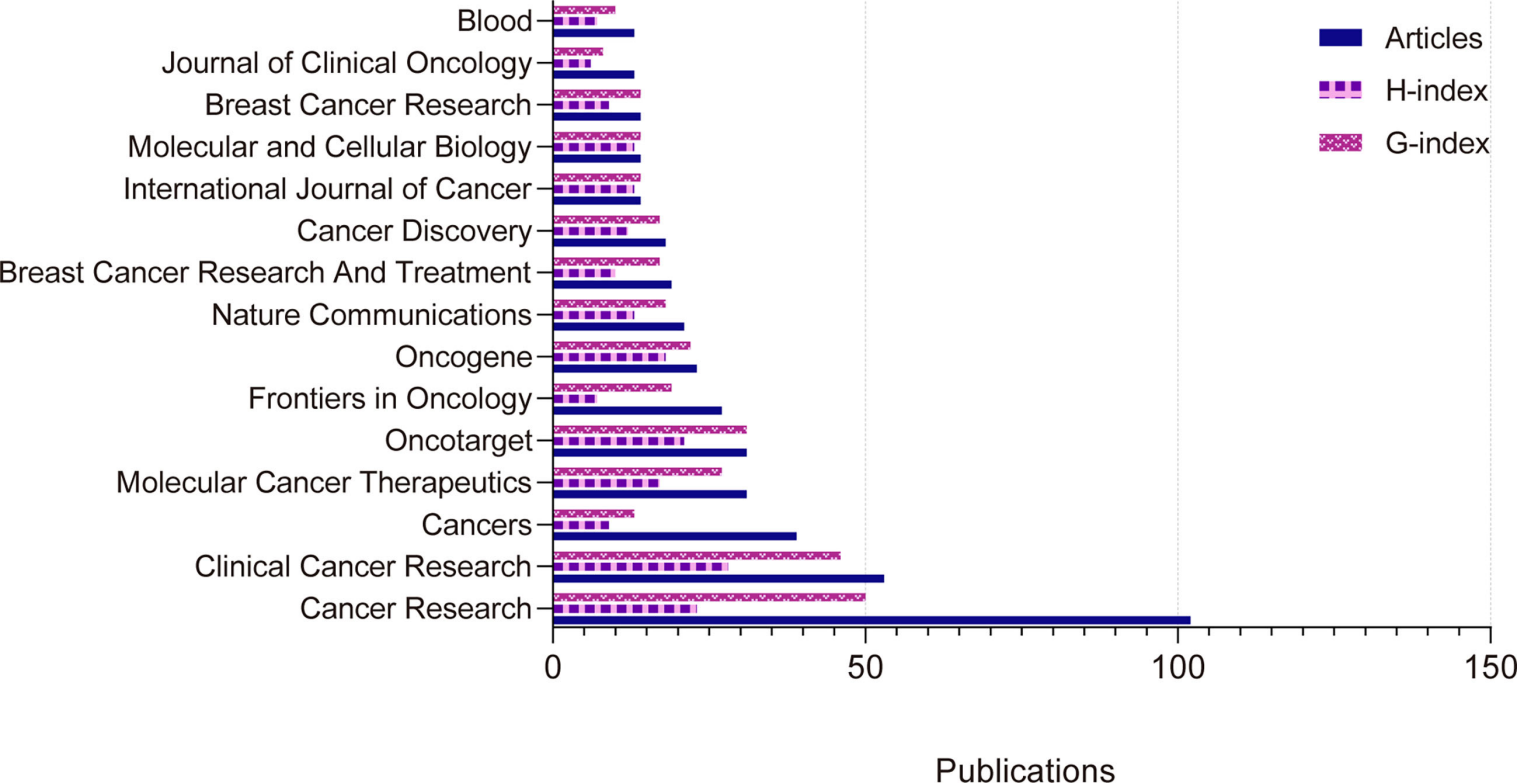
Fifteen journals in the core field of resistance to CDK4/6 inhibitors according to Bradford’s Law in Web of Science Core Collection.

According to Bradford’s Law (25), there are 15 core journals and 372 non-core journals in the field of CDK4/6 inhibitor resistance research in the core collection of Web of Science. (**Figure 2**) Among these 15 journals, Cancer Research had the highest number of articles, with 102, Clinical Cancer Research with 53, and Cancers with 39. The H-index and G-index are calculated for each journal. Impact factors and JIF quartile are counted by the Journal Citation Reports 2021. (**Table 1**) The H-index is the total number of published papers that have been cited at least H times. The G-index is a derivative of the H-index, which was proposed to compensate for the inability of the H-index to translate highly cited articles, interpreted as having G articles with an average number of citations not less than G. Both indices are used to indicate impact as a calculated index. In PubMed, there are 18 core journals and 343 non-core journals according to Bradford’s Law. Clinical Cancer Research has published the most articles with 54 articles. The statistical results are shown in supplementary **Supplementary Table S1**. To respond as much as possible to the relationship between CDK46 inhibitor-resistant published articles and impact factor, we decided to use the number of articles on CDK46 inhibitor resistance published in each journal in a single year in 2021 as a percentage of the total number of articles published in that year as a single factor for correlation analysis with the 2021 Journal Impact Factor (JIF). The results showed that Total Citations was the factor with the highest correlation (coefficient = 0.23) with JIF. H-index (coefficient = 0.14), percentage of articles with CDK46 inhibitor resistance (coefficient = 0.13), and G-index (coefficient = 0.10) correlated well with the impact factor too (**Supplementary Figure S1**, **Table S2**).

**Table 1:** Fifteen journals in the core field of resistance to CDK4/6 inhibitors according to Bradford’s Law.

Journal	Articles	IF/JIF Quartile	H-index	G-index	Category
Cancer Research	102	13.312/Q1	23	50	Oncology
Clinical Cancer Research	53	13.801/Q1	28	46	Oncology
Cancers	39	6.575/Q1	9	13	Oncology
Molecular Cancer Therapeutics	31	6.009/Q2	17	27	Oncology
Oncotarget	31	NA/NA	21	31	Cell Biology/Oncology
Frontiers in Oncology	27	5.738/Q2	7	19	Oncology
Oncogene	23	8.756/Q1	18	22	Biochemistry& Molecular Biology/Cell Biology/Gentics & Heredity/Oncology
Nature Communications	21	17.694/Q1	13	18	Multidisciplinary Sciences
Breast Cancer Research and Treatment	19	4.624/Q2	10	17	Oncology
Cancer Discovery	18	38.272/Q1	12	17	Oncology
International Journal of Cancer	14	7.316/Q1	13	14	Oncology
Molecular and Cellular Biology	14	5.124/Q2	13	14	Biochemistry & Molecular Biology/Cell Biology
Breast Cancer Research	14	8.408/Q1	9	14	Oncology
Journal of Clinical Oncology	13	50.769/Q1	6	8	Oncology
Blood	13	25.476/Q1	7	10	Hematology

### Author

**Figure 3:**
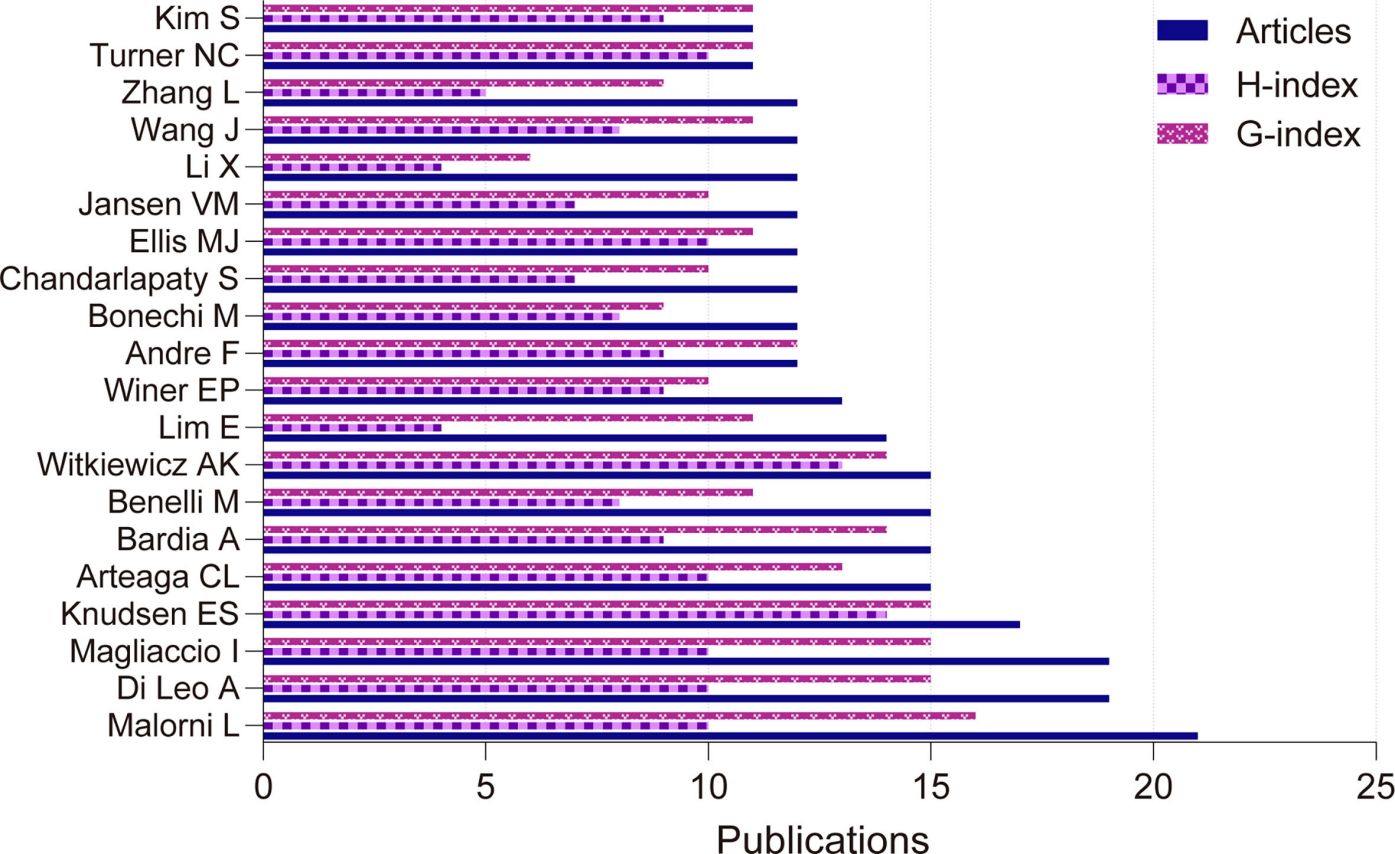
Most productive authors in Web of Science Core Collection.

Among the authors with the highest number of articles, (**Table 2**, **Figure 3**) the first author with 21 articles (1.6%) is Malorni L from Italy, who has the third highest H-index and the highest G-index. The highest H-index author is Prof. Knudsen ES from the United States. Prof. Turner NC has 2584citations and is tied for the third highest H-index and has an M-index of 1.429, indicating that Prof. Turner NC is a hot newcomer in the field of CDK4/6 inhibitor resistance research. M-index is calculated by dividing the H-index by the years since the first article was published in this dataset. The author with the highest number of published articles in PubMed, after removing statistical duplication due to renaming, was Bardia A with 16 publications. (**Supplementary Table S3**)

**Table 2:** The 20 most productive authors of resistance to CDK4/6 inhibitors.

Authors	Articles	Total Citations	H-index	G-index	M-index (Started Year)
Malorni L	21	395	10	16	1.111 (2014)
Di Leo A	19	388	10	15	1.111 (2014)
Migliaccio I	19	391	10	15	1.111 (2014)
Knudsen ES	17	1227	14	15	1.077 (2010)
Arteaga CL	15	1561	10	13	0.476 (2002)
Bardia A	15	766	9	14	1.286 (2016)
Benelli M	15	234	8	11	1.333 (2017)
Witkiewicz AK	15	966	13	14	1.083 (2011)
Lim E	14	134	4	11	0.800 (2018)
Winer EP	13	1453	9	10	1.286 (2016)
Andre F	12	1674	9	12	1.125 (2015)
Bonechi M	12	280	8	9	1.143 (2016)
Chandarlapaty S	12	551	7	10	1.167 (2017)
Ellis MJ	12	468	10	11	0.833 (2011)
Jansen VM	12	456	7	10	1.167 (2017)
Li X	12	52	4	6	0.400 (2013)
Wang J	12	337	8	11	0.500 (2007)
Zhang L	12	230	5	9	0.625 (2015)
Turner NC	11	2584	10	11	1.429 (2016)
Kim S	11	717	9	11	0.900 (2013)

### Institution

**Figure 4:**
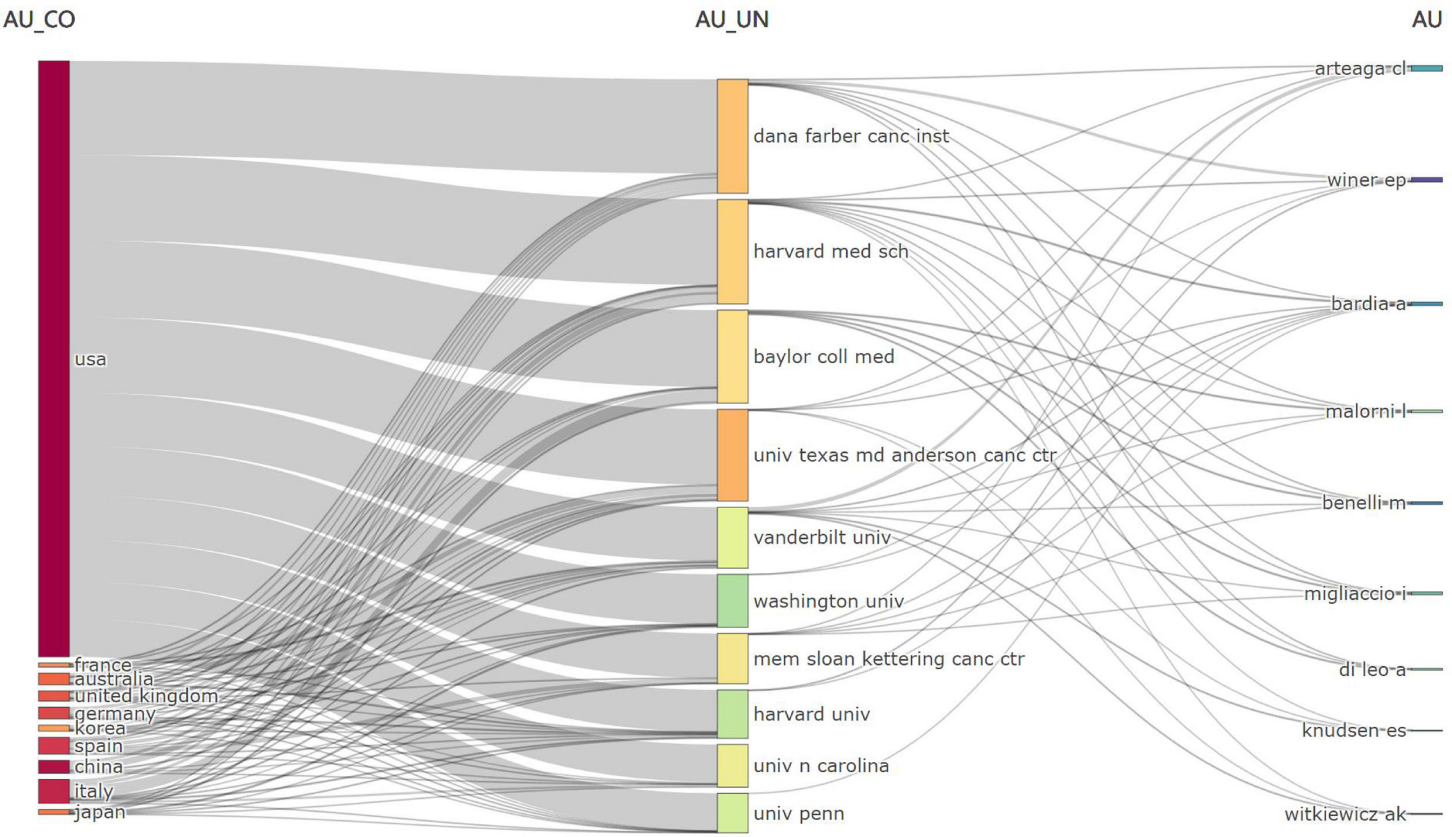
Three-Fields plot. (Country-Affiliation-Author).

**Figure 5:**
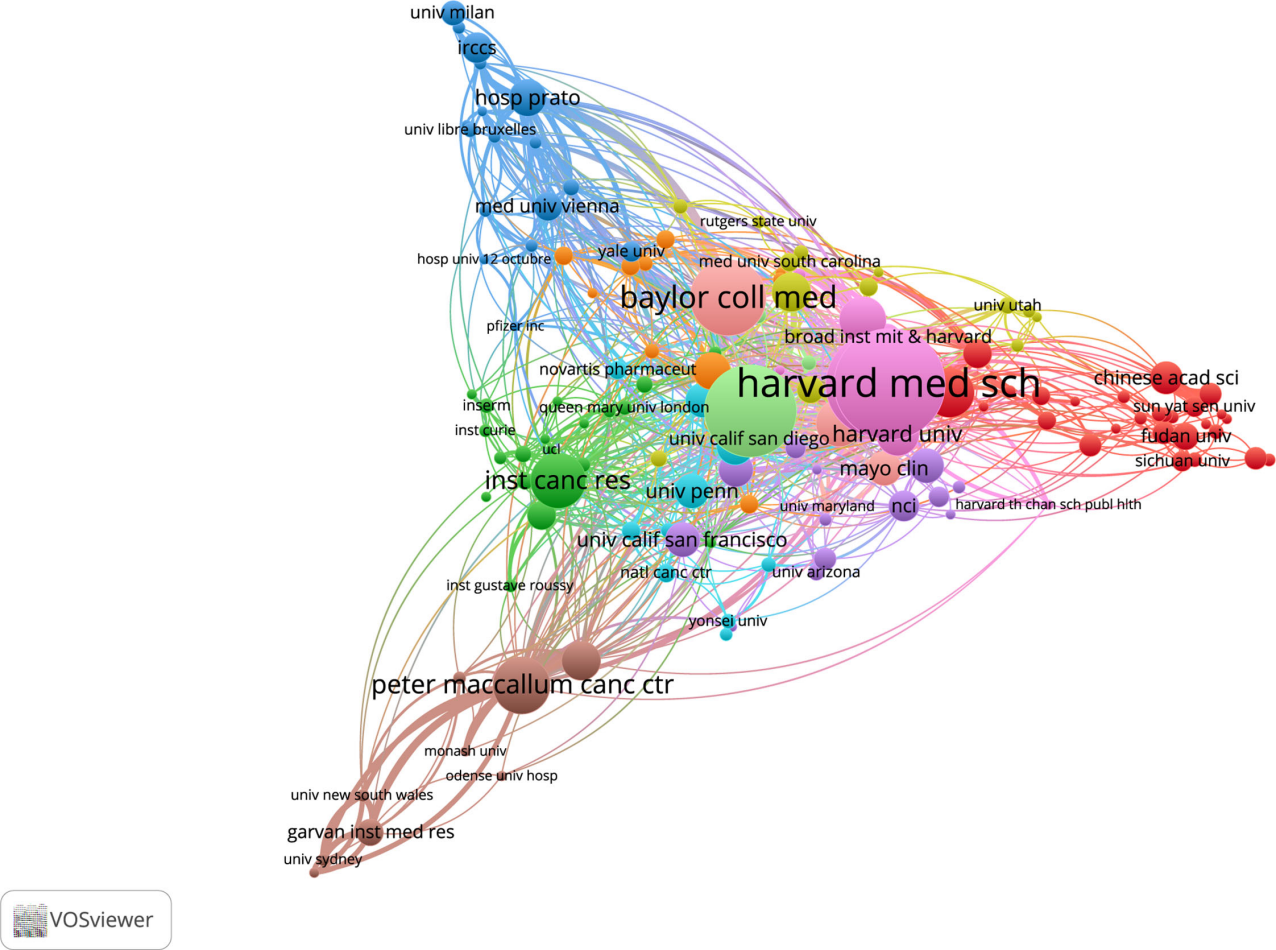
Most published institutions and their co-authorship network in Web of Science Core Collection.

In the Web of Science dataset, a total of 1735 institutions were involved in article publication, and 28 records did not contain data in the field being analyzed. The ten institutions with the most publications in the field of CDK4/6 inhibitor resistance have been listed in **Supplementary Figure S2**. Harvard University has the most articles in the dataset with 116 articles. A total of nine of the top ten institutions are located in the United States, with only the University of London being the UK-based publisher. In PubMed, a total of 2113 institutions were included in the statistics. Dana-Farber Cancer Institute authors appeared most frequently, with 380 occurrences. A total of eight of the top ten institutions are located in the United States, with The Institute of Cancer Research being the UK-based publisher and Peter MacCallum Cancer Centre being the Australia-based publisher. The Web of Science database article author’s institution is not duplicated, while the PubMed database counts the frequency of occurrence of article author’s institutions. Therefore, there is a difference in the number of institutional statistics between the two databases. To explore the collaborative relationships between different institutions, we draw a three-fields plot and use co-authorship to map collaborative networks. Only units with ≥5 articles are counted in the network map. (**Figures 4**, **5** and **Figure S3**)

### Country

**Figure 6:**
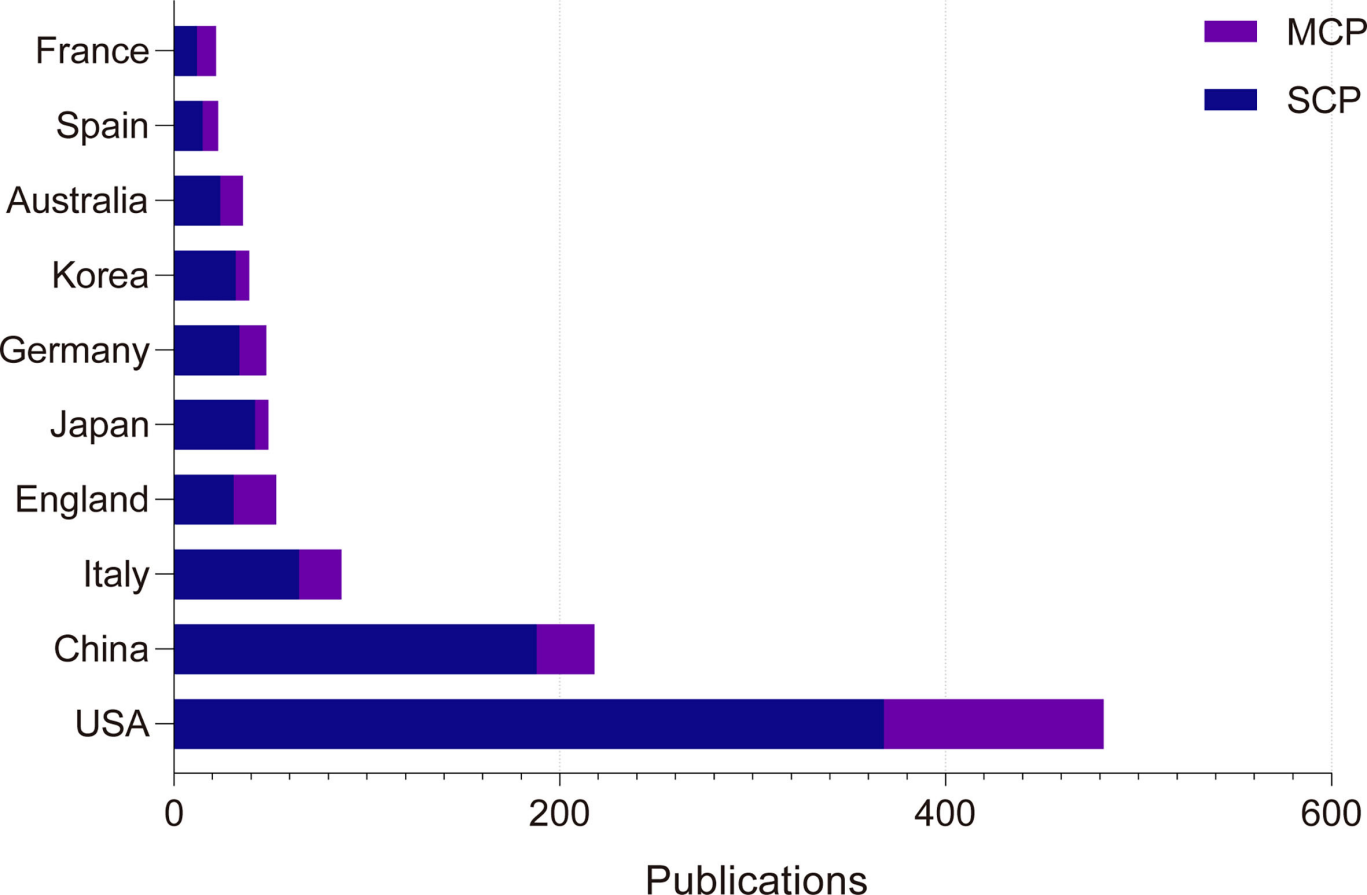
Top 10 productive countries’ SCP and MCP counts in Web of Science Core Collection.

**Figure 7:**
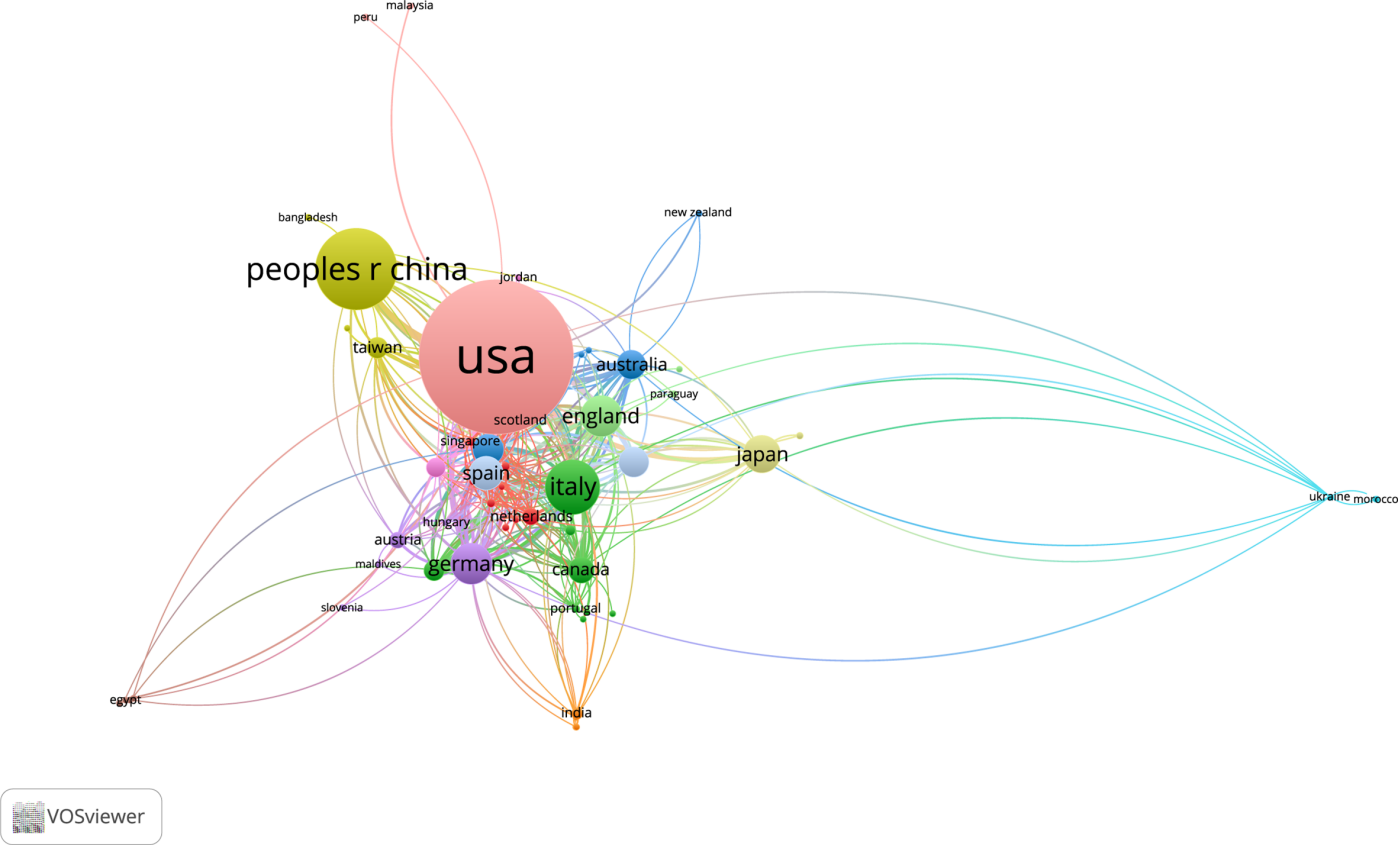
Network map of publications and collaborations of different countries in Web of Science Core Collection.

U.S. researchers were involved in the largest number of publications in the core collection of Web of Science, with 482 articles (37.45%). Although China ranked second in the number of participating publications, its average number of citations per article was low, with only 12.21. U.S. authors also published the most articles in PubMed, with 289 articles (25.73%).We classified articles as single-country publications (SCP) or multiple-country publications (MCP) based on whether the corresponding author is of the same or different nationality. The MCP rate in France is 45.5%, the highest of the ten most productive countries in Web of Science. The highest MCP rate among the top 10 countries in PubMed is the United Kingdom with 37.5%. (**Table 3**, **Figure 6**, **Supplementary Table S4**) The country relationship and country distribution were drawn based on the number of publications and co-authors. Of the 69 countries, 6 countries have no co-authorship ties with other countries. The remaining 63 countries are related to each other as shown in the figure. (**Figure 7**)

**Table 3:** Ten most productive countries of resistance to CDK4/6 inhibitors.

Countries	Articles	Average Article Citations	SCP^*^	MCP (MCP Ratio)^*^
USA	482	47.74	368	114 (23.7%)
China	218	12.21	188	30 (13.8%)
Italy	87	14.90	65	22 (25.3%)
England	53	56.68	31	22 (41.5%)
Japan	49	23.69	42	7 (14.3%)
Germany	48	20.38	34	14 (29.2%)
Korea	39	15.49	32	7 (17.9%)
Australia	36	25.61	24	12 (33.3%)
Spain	23	22.00	15	8 (34.8%)
France	22	26.18	12	10 (45.5%)

* single-country publication (SCP). multiple-country publication (MCP). Calculated from the corresponding author’s country only.

### Keywords

**Figure 8:**
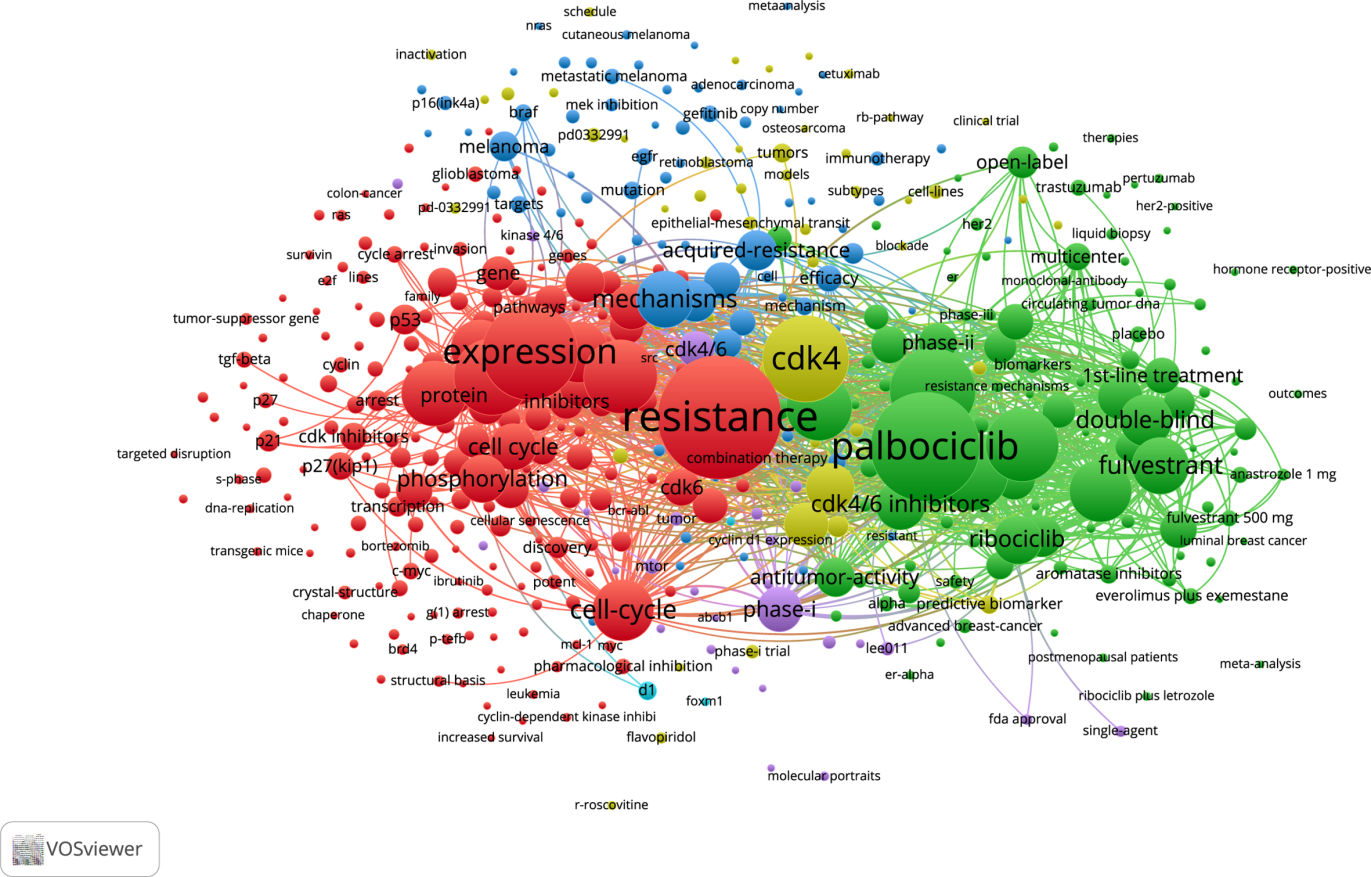
Co-occurrence network of all keywords in the dataset in Web of Science Core Collection.

**Figure 9:**
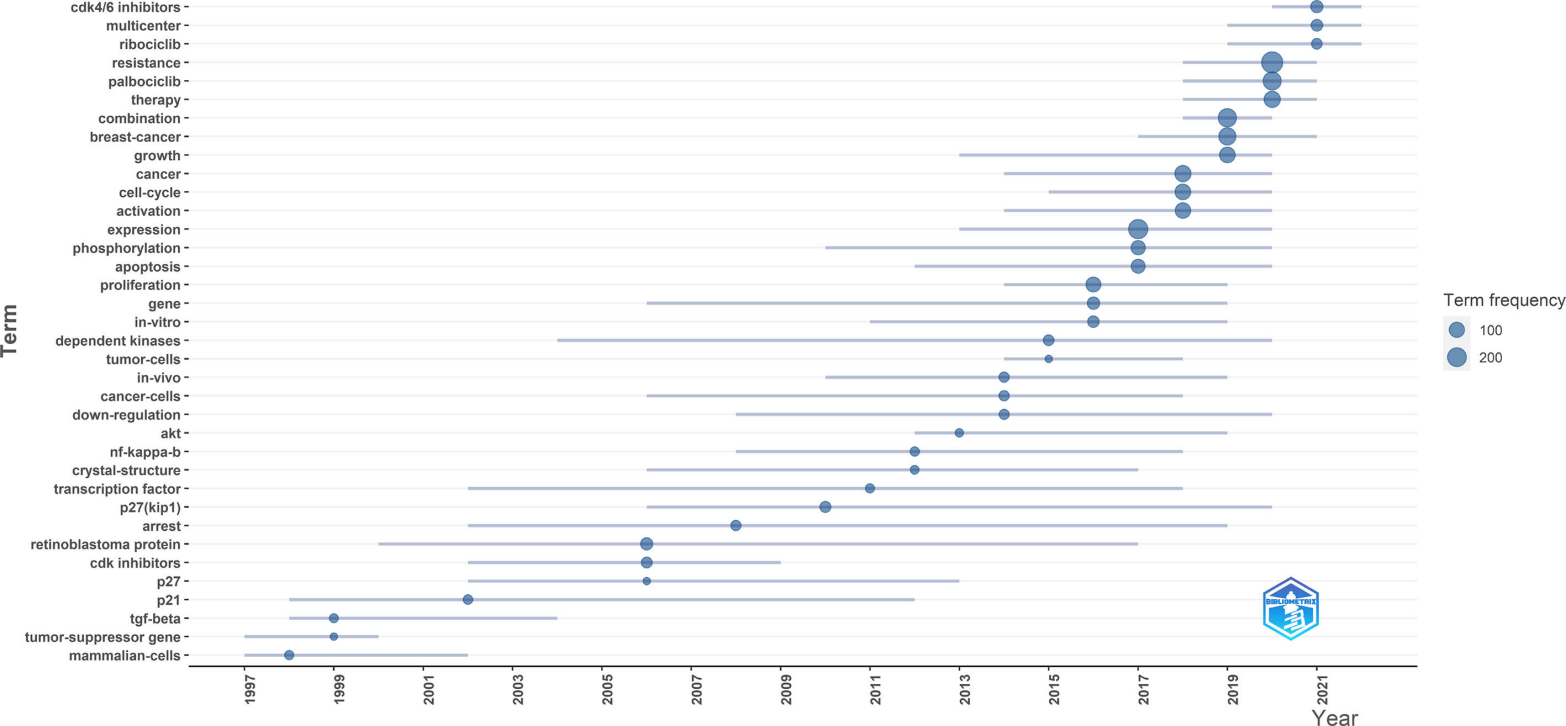
Trend topics of each year from 1996-2022 in Web of Science Core Collection.*Computed by Bibliometrix using Unigrams. Years that do not meet the criteria are not shown in the graph.

The frequency of occurrence and relationship of keywords can be highlighted by keyword co-occurrence. Keyword co-occurrence refers to the association between two words that appear in the same article. The stronger the co-occurrence relationship, the more it explains the topical direction of the study. Words with occurrences greater than 5 were counted in the co-occurrence statistics. A total of 476 words from 4026 keywords were included in the co-occurrence network mapping. (**Figure 8**) The same color represents the same clustering and the size of the circle represents the frequency of occurrence. At the same time, we conducted a topic trend mapping to see the hotspots of keyword appearances. (**Figure 9**) In the three years of peak citation, high-frequency words did not appear in 2000 and 2009. Proliferation appeared most frequently in 2016 with 88 times. The rest of the words with high frequency in 2016 were gene and *in-vitro*. The topic trend was filtered using keywords plus, with a minimum frequency of 10 occurrences of the word. Three words were selected each year.

**Figure 10:**
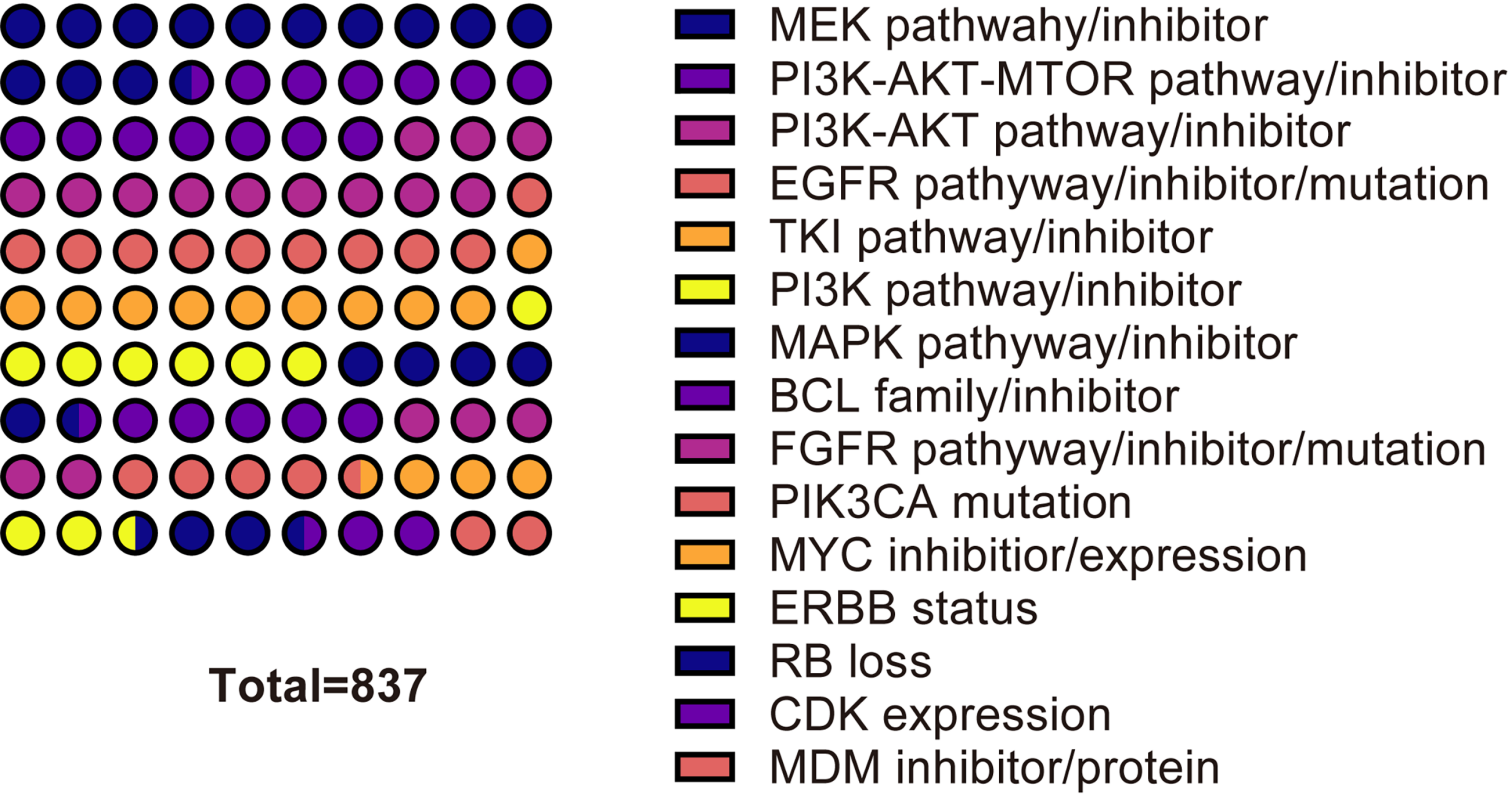
The most frequent mechanisms or treatment of resistance to CDK46 inhibitors in Web of Science Core Collection.

**Figure 11:**
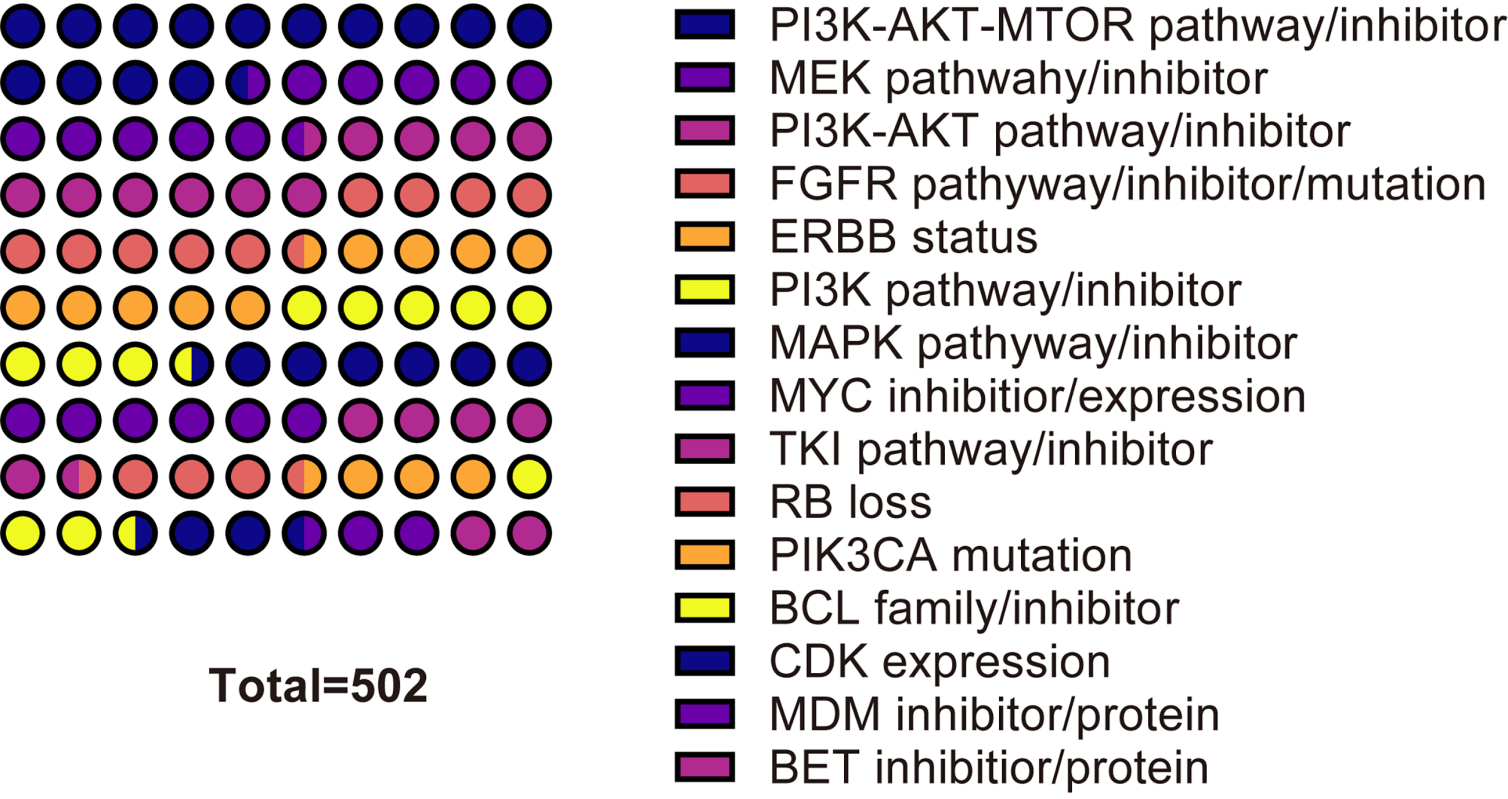
The most frequent mechanisms or treatment of resistance to CDK46 inhibitors in PubMed.

To understand the mechanism of CDK4/6 inhibitor resistance and post-resistance therapeutic measures to date, we performed metrological statistics on the literature highly related to the mechanism and post-resistance therapeutic methods studies. In the core collection of Web of Science, the highest frequency of 114 occurrences was associated with MEK inhibitors and related pathways. The frequency of PI3K-AKT-mTOR, a key pathway, also remained high. The frequencies of PI3K alone, PI3K-AKT, and PI3K-AKT-mTOR were 59, 99, and 113, respectively. Other elements that appeared with high frequency were mutations in EGFR-related pathways, EGFR inhibitors, TKI inhibitors, MAPK pathways and inhibitors, and so on. In PubMed, the highest frequency of occurrence was for the PI3K-AKT-mTOR pathway and related inhibitors. The 15 relevant mechanisms or treatments with the highest frequency of occurrence were selected. The top cited articles related to CDK4/6 inhibitors for the resistance mechanisms or therapeutics addressed in the chart have been listed. (**Figures 10**, **11**, **Supplementary Table S5**).

### Citations

Globally, the most cited article was published by Sherr, Charles J. in 2000 in the journal Cancer Research, “The Pezcoller lecture: Cancer cell cycles revisited”, with 1074 citations. However, the article has only 7 local citations in the entire CDK4/6 inhibitor resistance literature set. The second and third most cited articles describe the promising results of the PALOMA-3 trial and the results of *in vitro* experiments with palbociclib, with Local Citations/Global Citations (LC/GC) ratios of 22.96%(211/919) and 28.93(256/885), respectively. (**Table 4**) The LC/GC ratio can partially reflect the impact of the article in the field of CDK4/6 inhibitor resistance research. We further performed a bibliometric evaluation of original articles only from the WOS database. The most cited article globally was the PALOMA-3 trial in 2016. The article with the most local citations in the original article dataset was the results of the palbociclib *in vitro* trial in 2009 (**Supplementary Table S6**). The results show that in recent years the focus of research in the field of CDK4/6 inhibitor resistance has been mainly on palbociclib.

**Table 4:** The most globally cited articles on resistance to CDK4/6 inhibitors.

Title	First Author	Year	Journal	Local Citations (LC)	Global Citations (GC)	LC/GC ratio	Normalized Global Citations*
The Pezcoller lecture: Cancer cell cycles revisited	Sherr, Charles J.	2000	Cancer Research	7	1074	0.65%	7.01
Fulvestrant plus palbociclib versus fulvestrant plus placebo for treatment of hormone-receptor-positive, HER2-negative metastatic breast cancer that progressed on previous endocrine therapy (PALOMA-3): final analysis of the multicentre, double-blind, phase 3 randomised controlled trial	Massimo Cristofanilli	2016	Lancet Oncology	211	919	22.96%	13.82
PD 0332991, a selective cyclin D kinase 4/6 inhibitor, preferentially inhibits proliferation of luminal estrogen receptor-positive human breast cancer cell lines *in vitro*	Richard S Finn	2009	Breast Cancer Research	256	885	28.93%	7.47
Ribociclib as First-Line Therapy for HR-Positive, Advanced Breast Cancer	Hortobagyi GN	2016	The New England Journal of Medicine	182	821	22.17%	12.35
Treating cancer with selective CDK4/6 inhibitors	O’Leary B	2016	Nature Reviews Clinical Oncology	128	557	22.98%	8.38
A Cancer Cell Program Promotes T Cell Exclusion and Resistance to Checkpoint Blockade	Livnat Jerby-Arnon	2018	Cell	18	430	4.19%	12.26
An F876L Mutation in Androgen Receptor Confers Genetic and Phenotypic Resistance to MDV3100 (Enzalutamide)	Korpal M	2013	Cancer Discovery	5	384	1.30%	7.88
Cyclin-dependent kinases regulate the antiproliferative function of Smads	Matsuura I	2004	Nature	13	379	3.43%	5.26
AKT/PKB Phosphorylation of p21Cip/WAF1 Enhances Protein Stability of p21Cip/WAF1 and Promotes Cell Survival	Li Y	2002	Journal of Biological Chemistry	1	361	0.28%	3.86
Early Adaptation and Acquired Resistance to CDK4/6 Inhibition in Estrogen Receptor–Positive Breast Cancer	Herrera-Abreu MT	2016	Cancer Research	216	336	64.29%	5.05

* Normalized Global Citations: Total citations of the article/average citations of peer articles when compared to peer papers (papers published in the same year, in the same discipline, and also in the same literature type).

## Discussion

CDK4/6 inhibitors play an extremely important role in the field of cancer therapy, especially in breast cancer, and the study of their resistance mechanisms and the overview of their resistance status are the focus of attention worldwide. Based on data from PubMed and Web of Science, from 1993-2013, the number of publications was < 20 in all years.From 2014 onwards, the number of posts has increased rapidly compared to the previous. When the results of large-scale applications of different CDK4/6 inhibitors in trials such as PALOMA, MONALEESA, and MONARCH were published, studies and review analyses of CDK4/6 inhibitor resistance have been rapidly conducted. Among these included articles, the article-to-review ratio was 3.83 in Web of Science Core Collection, and 4.18 in PubMed.

The most frequently occurring word was resistance, with 309 occurrences, in 1278 included articles from Web of Science by full count. The 10 most frequent words in Web of Science were resistance, breast cancer, palbociclib, expression, cdk4, combination, cancer, abemaciclib, therapy, and apoptosis. In PubMed, The most frequently occurring words were human, cyclin-dependent kinase 4, female, animals, cyclin-dependent kinase 6, breast neoplasms, cell line, drug resistance, protein kinase inhibitors, and mice. It is inferred that the current research on CDK4/6 inhibitor resistance is mainly focused on palbociclib and abemaciclib. Abemaciclib, although approved by the FDA and EMA only in 2018, is the latest of the three drugs, but is rapidly becoming a hot subject of research. Palbociclib belongs to the pyridopyrimidine class, and is weakly basic in nature, with two pKa values of 3.9 and 7.4, respectively, and a calculated cLogP (log octanol-water partition coefficient) of 2.7 (26, 27). The IC50 of CDK4 and CDK6 in palbociclib were 0.011 and 0.016 µM, respectively (28). Abemaciclib is a ternary compound with pKa values of 3.80, 4.48, and 7.95. Due to the presence of three active metabolites of similar potency to abemaciclib, they have different IC50 values for CDK4 and CDK6. N-desethylabemaciclib (M2), hydroxyabemaciclib (M20) and hydroxy-N-desethylabemaciclib (M18) had IC50 values of 1.2nM and 1.3nM, 1.5nM and 1.9nM, 1.5nM and 2.7nM for CDK4 and CDK6, respectively (29). CDK4 is an important oncogenic factor in breast cancer and CDK6 plays a role in hematopoietic stem cell differentiation. abemaciclib inhibits CDK4 approximately five times more than CDK6, so abemaciclib is theoretically less hepatotoxic (30–33). CDK4/6 inhibitor resistance studies are mainly in the area of breast cancer, and the remaining application scenarios include melanoma, non-small cell lung cancer, gastric cancer, colon cancer, etc. The terms with high co-occurrence frequency are concentrated in both clinical application and drug experiments, such as combination, double-blind, therapy, phase-1, human, cell line, and so on. To understand the current mechanism and post-drug resistance therapies of CDK4/6 resistance research, we separately measured the non-review experimental articles both in the Web of Science Core Collection and in PubMed. The most frequently occurring related contents are MEK inhibitors and related pathways, PI3K-AKT-MTOR pathway or inhibitors, EGFR-related pathways, EGFR inhibitors, TKI inhibitors, MAPK pathways and inhibitors, BCL inhibitors, FGFR inhibitor or mutation, PIK3A mutation, MYC inhibitor or expression, ERBB status, RB loss, CDK expression, MDM inhibitor and so on. The increased expression of CDK6 may be due to the deletion of the *FAT1* gene (19, 34), and CDK2 overexpression may be due to the activation of the CCNE-CDK2 pathway (35, 36). The causes of PI3K/AKT/mTOR pathway activation are a hot topic of research, including *PTEN* loss of function, AKT1 alteration, PI3K mutation, etc (37–41). There are also some general directions regarding post-resistance therapeutic research, and the most discussed are PI3K/AKT/mTOR pathway inhibition, including PI3K inhibitors, mTOR inhibitors, AKT inhibitors, etc (37, 42–46). The abnormal activity of CDKs and the activation of the PI3K/AKT/mTOR pathway are both factors contributing to tumor growth, and the inhibition of both generally needs to be carried out simultaneously. Secondly, due to the overexpression of CDK2 after drug resistance, co-inhibition of CDK2 has also become a director of research (47–50). The rest of the mentioned therapies include MEK inhibition, FGFR inhibition, MDM2 inhibition, CDK7 inhibition, immunotherapy, etc (41, 51–56). CDK4/6 inhibitor resistance involves multiple pathways and multiple aspects. Its occurrence may not be uniform in time and space and needs to be analyzed specifically.

The main research in the field of CDK4/6 inhibitor resistance is concentrated in the United States. The United States has the highest number of articles published worldwide with 482 articles in the Web of Science and 289 articles in PubMed (each country is counted when the article has authors or institutions from different countries), and the average number of citations per article is 41.51 in Web of Science, which proves that the research value of the article is recognized by the international community. At the same time, the U.S. co-authored 23.7% of its articles with authors from other countries, a very high rate of international collaboration. China and Italy are the next countries in terms of the number of articles published, with218 articles from China and 87 from Italy in the dataset of Web of Science. While in PubMed, only 15.2% of U.S. authors collaborate internationally. We speculate that this may be the reason for the incomplete information provided by the PubMed database, as there are 290 articles where the authors’ countries are not attributed. In Web of Science, the average number of citations per article in China is 15.06 and the rate of international collaboration among authors is 13.8%, while the average citations and international collaboration of articles in Italy are not significantly different from those in the United State. Among the major publishing institutions, 80~90% are from the United States due to the statistics in both Web of Science and PubMed. The articles are concentrated in Harvard University, Harvard Medical School, University of Texas, Dana-Farber Cancer Institute, University of California, etc. According to the statistics, the US is the leading force in CDK4/6 inhibitor resistance research, but Europe, Asia, and other countries also play an invaluable role. This area has become a common focus worldwide.

Among the authors studying the issue of CDK4/6 inhibitor resistance, the most published author is Prof. Malorni Luca from the Hospital of Prato, Italy, with 21 articles. His earliest article on this area was a review published in 2014 in Current Opinion in Oncology, Cyclin-dependent kinase 4/6 inhibitors in breast cancer therapy (57), with an impact factor of 3.915 and 27 citations to date. Migliaccio Ilenia, also from Prato Hospital, contributed to this article as well. Malorni Luca appears alongside Prof. Migliaccio Ilenia in most of his published articles, covering basic research on gene expression that inactivates RB function, potential biomarkers of therapeutic resistance, and inhibition of CDK7, as well as occasional review articles on current resistance research or key breakthroughs (58–62). Also notable is Professor Turner Nicholas C from the UK. He was involved in the publication of the PALOMA-3 trial article in 2016, and the number of citations for 11 related articles to date has reached2584. At the same time, the H-index and G-index scores show that all 11 articles have a good research value and are recognized by peers and internationally. Among the top twenty authors, nine are from the United States, five from Italy, two from China, and one each from the United Kingdom, France, Australia, and Korea. It is worth noting that although the top 10 institutions do not include Italian national institutions, the five Italian authors among the top 20 authors are all from the Hospital of Prato, Italy.

Among the 15 most published journals counted, 10journals belong to the Q1 region according to the Journal Citation Report 2020, and 12 journals belong to the Oncology classification, thus concluding that CDK4/6 inhibitor resistance is a cutting-edge issue in oncology that has won the attention of researchers from all over the world. The most highly cited article worldwide was The Pezcoller lecture: Cancer cell cycles revisited published in Cancer Research in 2000. However, it only has 7 citations in the local dataset of CDK4/6 inhibitor resistance. The most highly cited article in the local dataset was PD 0332991, a selective cyclin D kinase 4/6 inhibitor, preferentially inhibits proliferation of luminal estrogen receptor-positive human breast cancer cell lines *in vitro*. Since palbociclib was the first CDK4/6 inhibitor approved by the U.S. Food and Drug Administration and European Medicines Agency, most of the research on drug resistance issues also started with palbociclib. Among the top 10 cited articles worldwide, the article with the highest LC/GC ratio is “Early Adaptation and Acquired Resistance to CDK4/6” published in Cancer Research in 2016 by Herrera-Abreu MT from the UK as the first author. The article investigates the mechanism by which acquired resistance to CDK4/6 inhibitors is generated by amplification of CCNE1 or deletion of Rb1 to bypass the cyclin D1-CDK4/6 complex and verifies that acquired resistance generated by CCNE1 amplification can be resolved by targeting CDK2 (63). The article has 216 citations in the current dataset and an LC/GC ratio of 64.29%, demonstrating that the efficacy of this article has been confirmed among experts.

## Limitations

The information in this dataset was obtained on 2022-07-31, and it was not possible to recount key articles in the field that were included after the date. Mechanisms or therapies related to CDK4/6 inhibition resistance that appear in only a single article are not shown in this paper, and there may be emerging ideas with significant discovery implications that are not counted in the statistics. The quality of published journals or articles cannot be directly evaluated by bibliometrics, and the value of specific articles should be judged only after systematic evaluation. The current work is only a general overview of the field as a whole.

## Data availability statement

The original contributions presented in the study are included in the article/**Supplementary Material**. Further inquiries can be directed to the corresponding author.

## Author contributions

YS designed the structure of this manuscript. JP collected the data and performed analysis. JP wrote the paper. HL revised the paper. All authors contributed to the article and approved the submitted version.

## Funding

This work was supported by construction and application of breast cancer panoramic database and biospecimen holographic library (Grant Number SHDC2020CR5005).

## Conflict of interest

The authors declare that the research was conducted in the absence of any commercial or financial relationships that could be construed as a potential conflict of interest.

## Publisher’s note

All claims expressed in this article are solely those of the authors and do not necessarily represent those of their affiliated organizations, or those of the publisher, the editors and the reviewers. Any product that may be evaluated in this article, or claim that may be made by its manufacturer, is not guaranteed or endorsed by the publisher.

## Supplementary material

Supplementary Figure 1 Pearson correlation coefficient.

Supplementary Figure 2 Ten institutions with the most publications in the field of CDK4/6 inhibitor resistance.

Supplementary Figure 3 Co-authorship of all the institutions in Web of Science Core Collection.

Supplementary Table 1 18 core journals according to Bradford’s Law in PubMed.

Supplementary Table 2 Pearson correlation coefficient.

Supplementary Table 3 10 most productive authors in PubMed.

Supplementary Table 4 10 most productive countries in PubMed.

Supplementary Table 5 Article list of mechanism or treatment of CDK4/6 inhibitor resistance.

Supplementary Table 6 10 most globally cited articles on resistance to CDK4/6 inhibitors in an original article dataset (excluding reviews, commentaries, editorials, etc.).
