# Supplementary figures and images for: CDK4/6 inhibitor resistance: A bibliometric analysis
Source: Front Oncol. 2022 Dec 1;12:917707. doi: 10.3389/fonc.2022.917707 (PMC9752919; doi:10.3389/fonc.2022.917707)

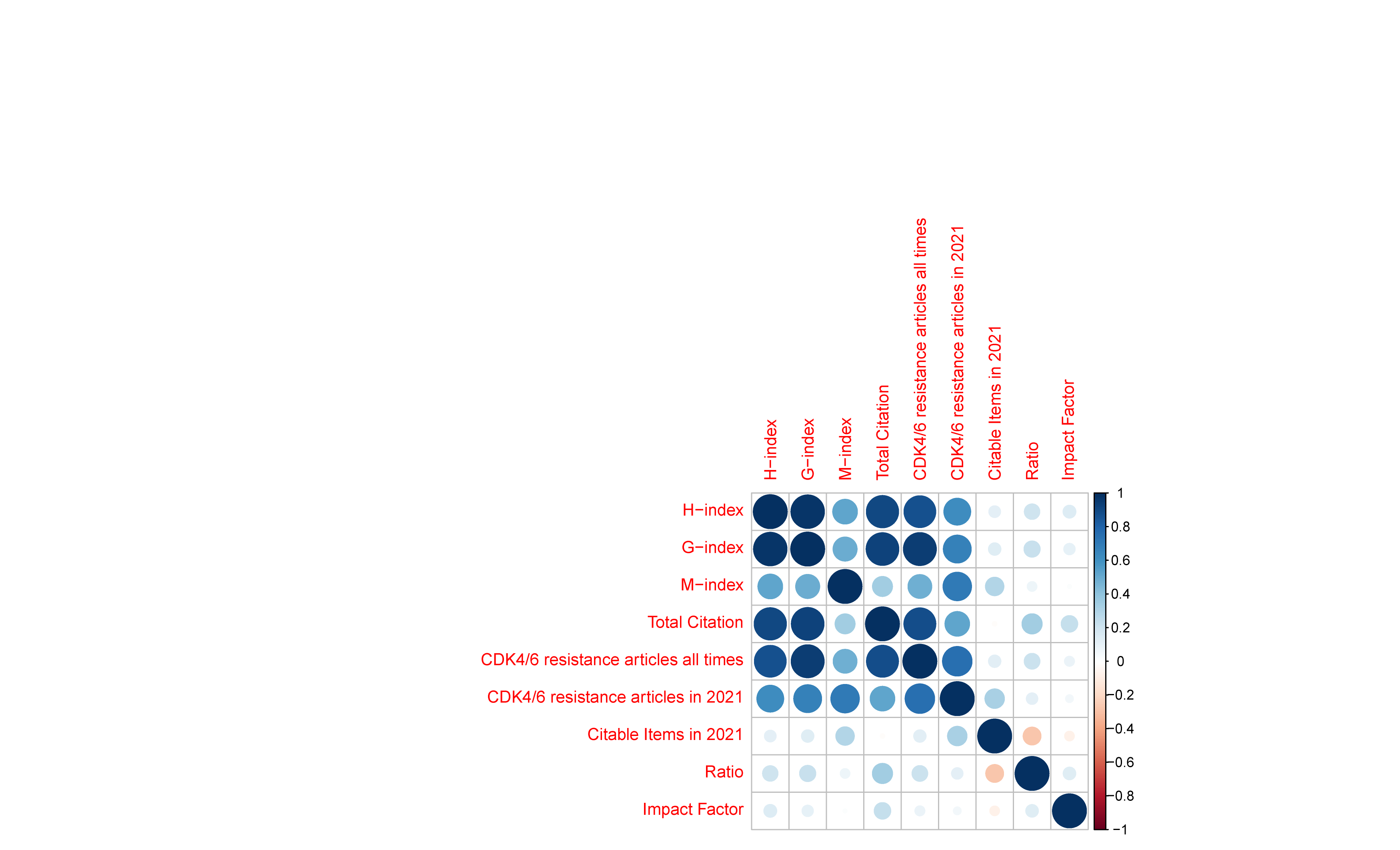

Supplement: Supplementary Figure 1 — Pearson correlation coefficient. [file Image_1.tif]

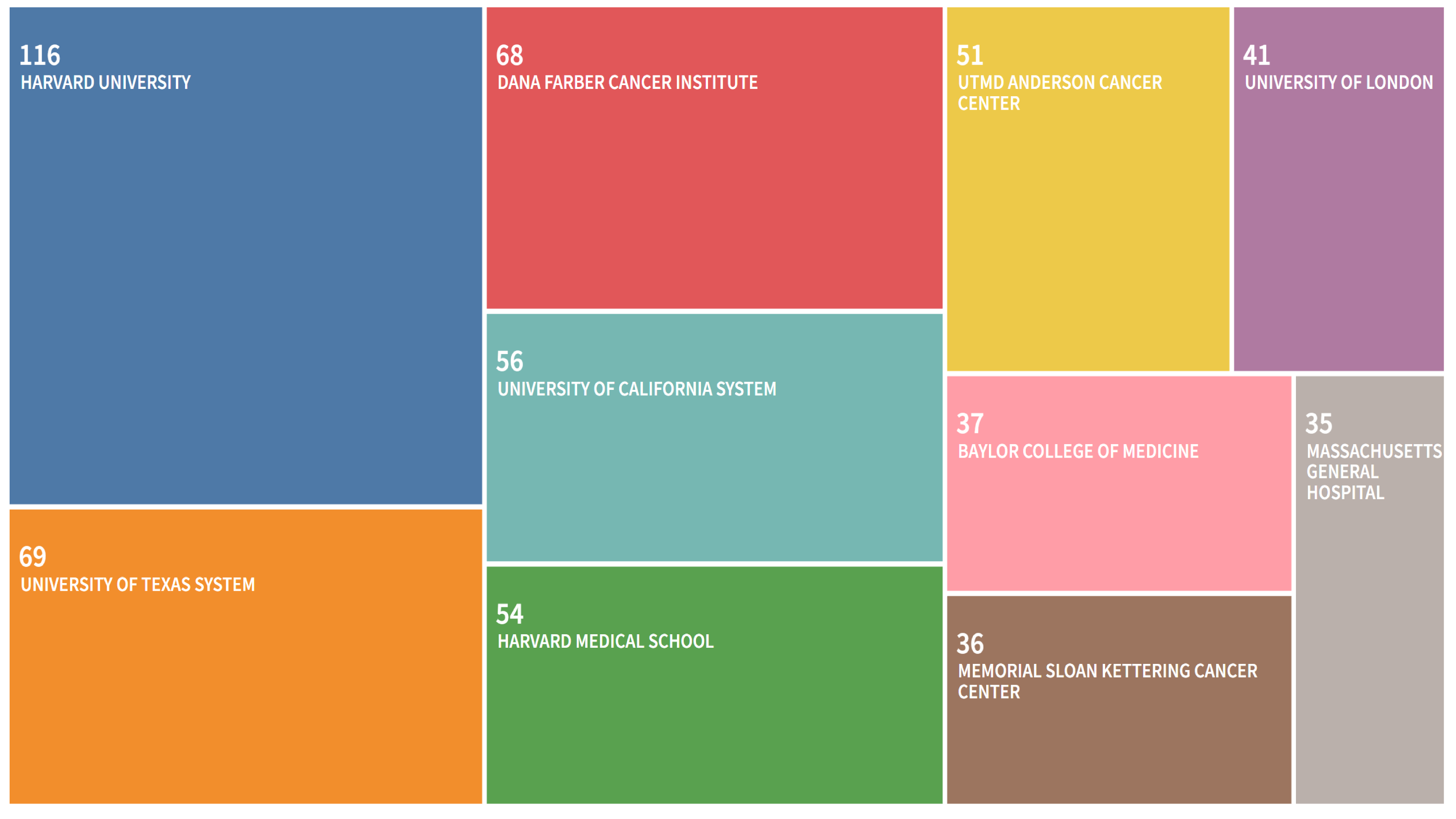

Supplement: Supplementary Figure 2 — Ten institutions with the most publications in the field of CDK4/6 inhibitor resistance. [file Image_2.tif]

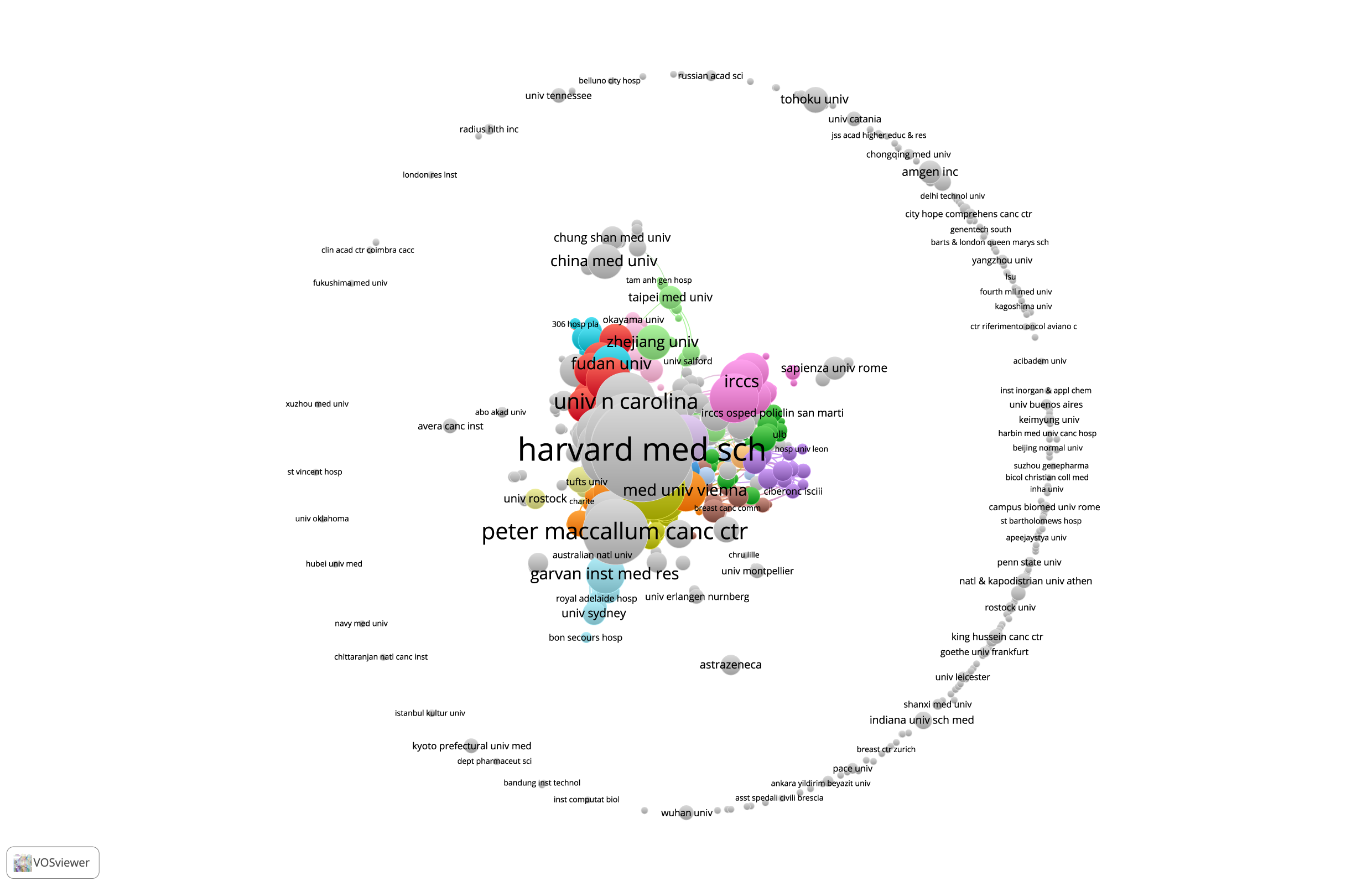

Supplement: Supplementary Figure 3 — Co-authorship of all the institutions in Web of Science Core Collection. [file Image_3.tif]
